# Do Lambs Perceive Regular Human Stroking as Pleasant? Behavior and Heart Rate Variability Analyses

**DOI:** 10.1371/journal.pone.0118617

**Published:** 2015-02-25

**Authors:** Marjorie Coulon, Raymond Nowak, Julie Peyrat, Hervé Chandèze, Alain Boissy, Xavier Boivin

**Affiliations:** 1 INRA, Unité Mixte de Recherche 1213 Herbivores, Site de Theix, 63122 Saint-Genès-Champanelle, France; 2 Clermont Université, VetAgro Sup, Unité Mixte de Recherche Herbivores, BP 10448, 63000 Clermont-Ferrand, France; 3 INRA, Unité Mixte de Recherche 85 Physiologie de la Reproduction et des Comportements, 37380 Nouzilly, France; 4 CNRS, Unité Mixte de Recherche 7247, 37380 Nouzilly, France; 5 Université François Rabelais de Tours, 37041 Tours, France; 6 Institut Français du Cheval et de l’Equitation, 37380 Nouzilly, France; Université de Montréal, CANADA

## Abstract

Stroking by humans is beneficial to the human-animal relationship and improves welfare in many species that express intraspecific allogrooming, but very few studies have looked at species like sheep that do not express such contact except around parturition. This study investigated the way lambs perceive regular human tactile contact using behavioral and physiological responses. Twenty-four lambs were reared and bucket-fed in groups of four. All were stroked daily by their familiar caregiver. At 8 weeks of age, the lambs were individually tested in their home pen but in a 1×1m open-barred pen after a 15h period of habituation to physical separation from peers while remaining in visual and auditory contact. Half of the lambs received stroking by their caregiver for 8min and half were exposed to their caregiver’s immobile presence. Heart rate and heart rate variability were recorded and analyzed by 2-min slots over the same interval based on three measures: mean heart rate value (HR), root mean square of successive differences (RMSSD) and standard deviation of all intervals measured between consecutive sinus beats (SDNN). Behavioral responses (ear postures of the lamb and time spent in contact with the familiar caregiver, on the knees of the familiar caregiver, and moving) were recorded throughout the test. Lamb HR decreased continuously while in the presence of their caregiver. Lambs being stroked showed slower HR and higher RMSSD which reflected positive emotional states compared to lambs left unstroked. All behavioral variables were highly correlated with the main component axis of the PCA analyses: the more the animals stayed in contact with their caregiver, the less they moved and the more their ears were hanging. This first component clearly differentiates lambs being stroked or not. Behavioral and physiological observations support the hypothesis that gentle physical contact with the caregiver is perceived positively by lambs.

## Introduction

Beneficial effects of intra or interspecific (i.e. human/animal) gentle stroking on behavior and physiology have mainly been explored in primates, rodents or dogs (for review, [1,2,3]). Heart rate and blood pressure decrease, oxytocin releases and relaxation states have been observed in animals during stroking. Variations in similar physiological biomarkers are also observed in the human performing stroking, thus showing a reciprocal beneficial effect [4]. In farm animals such as cattle, frequency of gentle interactions is significantly correlated to short avoidance distance of cows to humans and to improved milk production. Bertenshaw et al. [5] observed that a positive human-cow relationship can develop through tactile contact. Tactile contact, provided at least 5 min per week in the weeks before calving for a total of four hours to cows aged 6 to 24 months reduced fear of humans and promoted ease of handling during milking (e.g. less stamping, kicking, faster milk let-dow). More precisely, stroking mimicking intraspecific allogrooming, in particular when applied under the neck, has been shown to elicit a calming, anti-stress response including relaxed body postures, increased approach towards humans, and lower heart rate (e.g. in dairy cattle [6,7]). Stroked dairy cows were also less agitated during human veterinary interventions such as rectal palpation [8]. Recent studies in beef cattle showed that gentle stroking reduced avoidance responses and slaughter stress when provided at an early age but not just before slaughter [9,10].

This beneficial effect of human gentling is observed mainly in species that show intraspecific allogroming in adulthood. However, there is more debate over the effect of human gentling on animals, for which intraspecific tactile stimulation is mainly limited to the few hours following parturition (for example in horses [11] or in sheep [12]). On one hand, Feh and Demaziere [13] observed that human grooming reduces heart rate, but they did not record behavioral responses to humans. On the other hand, Henry et al. [11] observed very little positive effects of human tactile stimulation on foal behavior and concluded that stroking as a positive reward is probably acquired at later stages through other human-given rewards (i.e. food). In addition, results from Sankey et al. [14] suggested that tactile contact in horses was not perceived sufficiently positively to enable bonding or enhance learning. The importance of gentling in farm herbivore ungulates is thus a subject of debate among farmers and in the scientific community which is increasingly exploring the issue [11,15].

Sheep (*Ovis aris*) are quite similar to horses in terms of early development and intraspecific tactile stimulations. Their neonates are precocial, and they show very little intraspecific tactile stimulation other than when ewes actively lick their lambs during the first hour post-parturition [16] due to their attraction for the amniotic fluid. In sheep management, lambs are commonly reared without their dams for a variety of reasons—big litters, weak lambs, insufficient milk production, poor maternal abilities—in which case they are reared in groups of peers and trained by a human to drink from a milk-feeder. This longstanding conventional practice has been shown to facilitate the establishment of a strong bond with humans who can at least partly replace the maternal figure in terms of care provision due to a combined effect of early separation from the mother (loss of the attachment figure) and daily gentle contact with a human [17,18].

Tallet et al. [18,19] showed that artificially-reared lambs receiving human tactile contact daily during the first weeks of age expressed a much higher affinity for their caregiver than those receiving either only visual contact or no visual or tactile contact. However, these studies did not show whether such affinity stems from familiarization to human contact *per se* or to the ‘positive’ nature of these interactions. Recent studies have explored behavioral indicators of positive emotion in sheep [20–23]. Presumed positive situations (feeding, human grooming after voluntary approach from the sheep), in contrast to more negative situations (social separation, suddenness, unfamiliarity), are characterized by few ear posture changes, low proportions of asymmetric ear postures and high proportions of axial ear postures.

Heart rate variability, which is a measure of the balance between sympathetic and vagal activity [24], is another good indicator of emotion in animals. Reefman et al. [23] observed long mean inter-heartbeat intervals and high heart rate variability during human grooming compared to neutral (sheep standing in their home pen) or negative (social isolation) situations. Behavioral and physiological responses were also significantly correlated. However, work by Reefman et al [23] was inconclusive on how the sheep perceived human stroking as the effects of grooming and human presence were confounded. They did not rigorously evaluate the human grooming situation *per se* (six of the 15 experimental animals did not accept human contact) compared to passive human presence (where animals can nibble the clothes) nor did they provide any information on the quality of the human-sheep relationship and previous interactions, particularly in terms of food reward.

If stroking is perceived positively by lambs regularly stroked in early age, we hypothesize that once the human-animal relationship is established, stroking should trigger a larger decrease in heart rate, stronger activation of the parasympathetic system, and more calmed behavioral expressions compared to simple passive human presence.

## Methods

### Ethics Statement

The experimental protocol (Authorization N°CE04-11) using animal and human subjects was reviewed and approved by an institutional review board. All human subjects were employees of the research institute and they were informed and gave their written consent in their work contract to participate in experimentations on animal behavioral studies. The ethics committee, named “Comité Régional d’Ethique en Matière d’Expérimentation Animale Région Auvergne”, reviewed and approved both human and animal part of the protocol.

### Animals

**Fig 1:**
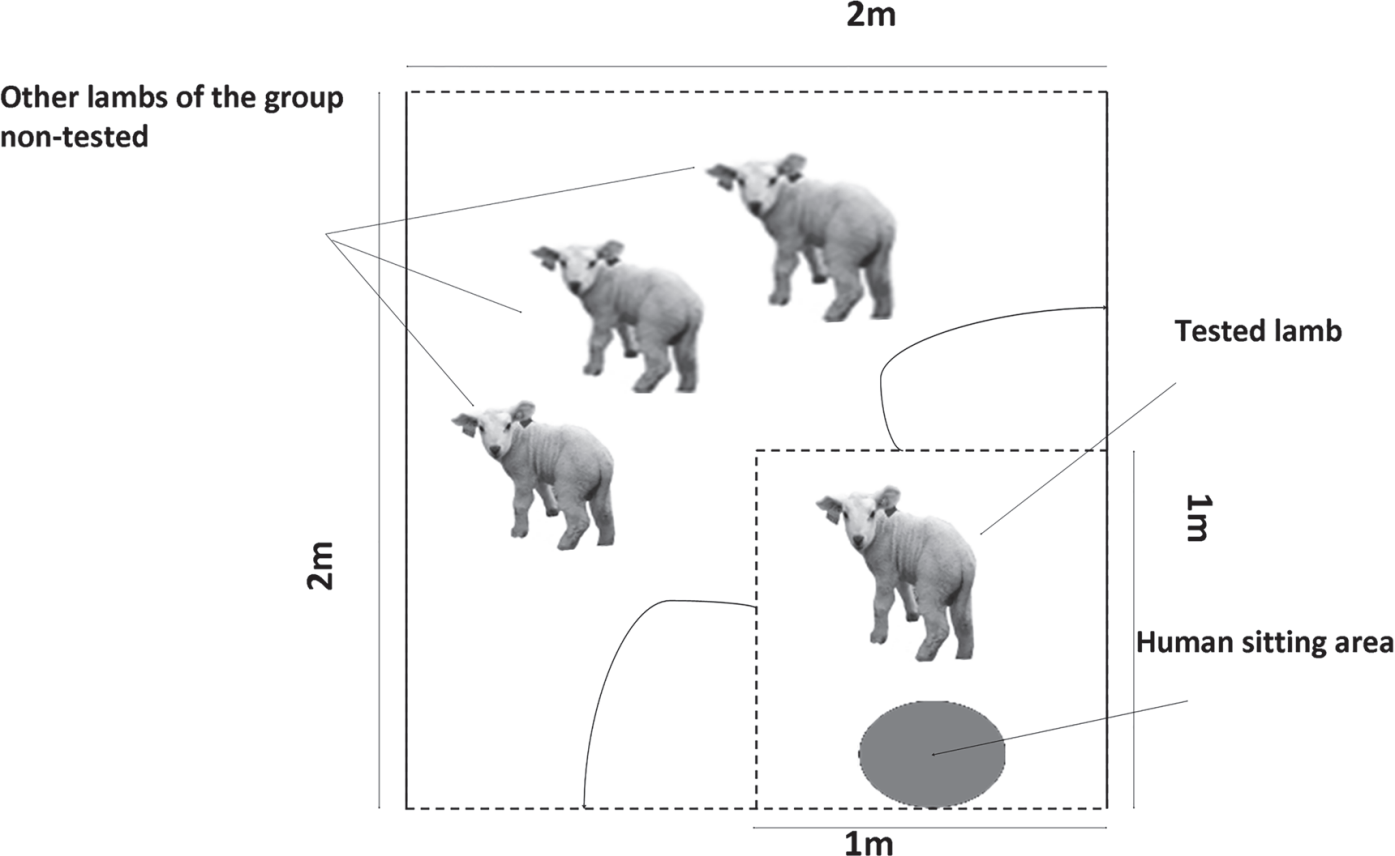
Diagram of the home pen with the small pen where the 8-week-old tested lamb was isolated to test behavioral and cardiac responses to tactile contact (solid lines: opaque wooden fences and dotted lines: open-barred fences). The barriers of the 1x1 m testing area could be open as mentioned by the curved arrows.

This experiment used 24 female *Romane* lambs born within one week. The lambs were ear-tagged and weighed at birth, left with their mothers for 12 hours in order to ensure adequate ingestion of colostrum, then separated from them to be reared artificially as per standard farming practices. Immediately after separation, the lambs were randomly allocated into groups of four and housed in a straw-bedded home pen (2×2 m, Fig. 1). A single stockwoman, wearing blue coveralls, spent the first two days training the lambs to suckle from a bucket fitted with four teats (i.e. six training sessions per pen, even if the lambs had already learned to suckle). Each lamb underwent three 30 sec—training session, after which they suckled without any further assistance. The lambs were fed a combination of milk replacer and water (250 g of powder/liter of ‘Cremagneau-Chevreau’ from Nutrilia-Sanders) over two meals given at 08:00 and 17:00 h. The bucket was introduced from outside the pen and left in the pen for 45 min. Opaque wooden fences separating the groups meant that the lambs had no visual or direct physical contact between groups but could still hear lambs from other pens. All other husbandry procedures (bedding, preparing milk distribution, veterinary treatment, etc.) were performed by another male stockperson wearing green coveralls. Two lambs had to be removed from the experiment due to health issues.

### Additional human-lamb contacts

Additional contacts were performed to facilitate the establishment of a bond to a human. They were provided by the same woman who was qualified as the “familiar caregiver”. Tactile stimulation (i.e. stroking) was provided daily over almost all of the experimental period. Before the start of the experiment, the familiar caregiver has trained to harmonize speed of stroking (between 40 and 60 strokes per minute) and to use a standardized medium pressure. Tactile stimulation was provided at least either 30 min before or 30 min after the green-coveralled stockperson brought the milk bucket so as to avoid any association between food and stroking. In the first week, the familiar caregiver stayed with each group for a 6-min session three times a day for 6 consecutive days. She entered the pen, crouched down, and one-by-one caught each lamb and petted it over the whole body surface including the back legs for 30 s while talking softly. In the following four weeks, tactile stimulation was provided three times a day for five consecutive days, and then once a day until two months of age.

The lambs were also habituated to another person whose role was to carry them to the experimental pen for habituation to and fitting of the heart rate monitor. This person (female) wore a white lab coat and stayed in front of each home pen for 3 min per day during handling sessions without making any attempt to attract the attention of the lambs and without making any tactile contact. In summary to help animals discriminate people via the color of their clothing [25], experimenters wore blue clothes for stroking, white clothes for leading animals to tests and green clothes for normal husbandry procedures. All the experiments were carried out at the UERT-Intrabois experimental farm located at INRA Clermont-Ferrand–Theix, France.

### Affinity to humans by 3-week-old lambs: behavioral response

Testing our hypothesis required the lambs to develop a strong level of affinity for their familiar caregiver. Boivin et al. [17] and Coulon et al. [26] showed a durable affiliative response of artificially lambs to humans when they had been stroked (at least up to 8 weeks of age). Accordingly, in the first step of this study and referring to their protocol, the level of lambs’ affinity towards their familiar caregiver was checked at 3 weeks of age [26]. All the tests were video-recorded (using a hard disk, handheld, JVC Everio GZ-MG255), and behavior was analyzed later on using The Observers 5.0 software. The day before, the person wearing the lab coat carried each group of lambs from the home pen to the test pen (10 to 20 m away). The lambs were left with their own group for 1 hour to acclimate to the test pen (4×1 m). The test pen was fenced with 2 m-high wooden panels and divided into four 1 m² zones by white lines painted on the floor. On the day of the test, the familiar caregiver brought one lamb into the test pen via the central door. The test was run in three 2-min phases. In phase 1, the animal was left alone. In phase 2, the familiar caregiver entered the pen via a side door located at each end of the corridor (the right-side door for one half of the lambs and the left-side door for the other half) and crouched down in the middle of either end of the pen (contact area). During this phase, if the lamb approached the contact area, the familiar caregiver slowly extended her arm towards the lamb. If the lamb made contact with the hand, the familiar caregiver began to stroke it. In phase 3, the familiar caregiver left the pen and the animal was again left alone for 2 min. Time spent in the contact area (with or without the familiar caregiver), total number of vocalizations, and number of zones crossed were recorded for each phase of the test. This test was repeated on the following day with the same animal in order to test the consistency of the lambs’ response to their familiar caregiver.

### Behavioral and cardiac responses to tactile contact by 8-week-old lambs

The same 22 lambs were re-tested at 8 weeks of age over a two-week-period. It was necessary to wait until such age since both the sympathetic and parasympathetic systems had been fully developed [24]. We compared the responses of half of the lambs caught and stroked for 8 min by their familiar caregiver (as during the three weeks of stroking sessions) to the responses of the other half simply exposed to the familiar motionless caregiver. Lambs were randomly allocated to each treatment balanced within their birth dates and home pens. Within-group order was balanced according to the treatment. Over the two days before the test, the lambs were habituated to wearing on their back a 120 g heart rate monitor fixed in an elastic belt strapped around the animal’s thorax (Transmitter ZB-512P, Nikon Kodhen, Japan) while in their home pen for 1 hour per day. As individual responses were targeted and conspecifics interaction could interfere with heart rate signal recording, physical separation from peers was needed to perform the test. Therefore, at 17:00 h the day before, the tested animal was put in a small pen built inside its home pen with open-barred fencing (1×1 m, one pen per group; Fig. 1). Such small pens (around 1m²) have currently been used in sheep to observe their emotional reactivity [27,28]. The lambs were familiar to this part of their home pen, as the fences were usually left open, but they were not accustomed to social separation. While isolated, the lambs still had tactile (with their nose), auditory and olfactory contact with peers. In addition, testing in the home pen removed confounding factors such as human handling, transportation and exposure to novel environment.

On the day of the test, lambs were tested in the morning only, at least 30 min after milk feeding, and over a period of several days as only four lambs per day could be tested (two of each modality). Each day, one lamb per experimental pen was tested. The heart rate monitor was put on the lamb by a familiar experimenter (wearing the white labcoat). Cardiac activity was recorded via two adhesive electrodes placed on the right shoulder and on the left axillary region of the lamb which had been shaved two days before. The electrodes were connected to a transmitter fixed in an elastic belt strapped around the animal’s thorax in a way that did not impair its movements. A receiver (Lifescope 6, Nikon Kodhen, Japan) was installed outside the experimental pen to capture the signal sent by telemetry. This receiver was connected to a computer running a data processing system (Powerlab, ADInstruments, UK). After 15 min, the familiar caregiver entered the small pen, crouched down and remained immobile. This procedure was standardized and lasted for two minutes. In half of the cases, a lamb was stroked for 8 min by first putting it on her knees in the same manner as during the training sessions. The lamb was free to move away from the familiar caregiver. For the other half, a lamb was exposed to the familiar caregiver’s immobile presence for the same duration, without being stroked.

Heart rate was recorded during last 2 min of the first phase (lamb alone) for each subject, and during the 8 min of human presence. The following variables were analyzed by 2-min slots using Chart software (version 3.6.8, HRV extension, ADInstruments, Australia): mean heart rate value (HR), root mean square of successive differences (RMSSD) and standard deviation of all RR (or NN = time intervals between consecutive heart beats) intervals measured between consecutive sinus beats (SDNN). Time-domain HRV variables such as SDNN reflect both sympathetic and parasympathetic activity, and RMSSD more specifically reflects parasympathetic activity. These measurements allow the reliable assessment of sympathovagal balance [29] that reflects the activity of the autonomous nervous system.

**Fig 2:**
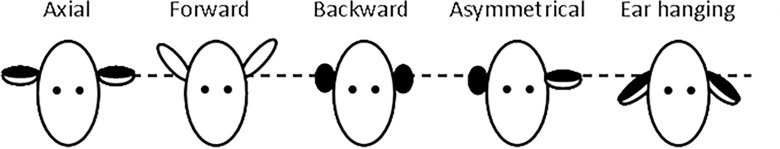
Position of the ears recorded in the 8-week-old tested lambs. Position of the ears in relation to frontal plane of the head and the orientation of the auricles observed in front of the animal: ear in axial posture, forward posture, backward posture, asymmetrical posture and ear hanging (adapted from [31]).

The test was video-recorded and the following behavioral variables were recorded: the latency to approach the familiar caregiver when she entered the small pen, then during 8 min the percentage of time spent in contact (any part of its body) with her, on her knees of the familiar caregiver, and moving. Ear postures of the lamb were also recorded every 15 sec during 8 min as in Reefmann et al. [30], and expressed as percentage of total observations: ear hanging, ear in axial posture, forward posture, backward posture and asymmetrical posture (Fig. 2). Attempts to escape the pen and vocalizations were also recorded when the familiar caregiver entered the pen and during the 8 min.

### Statistical analysis

All data was analyzed using SAS software (version SAS 9.1., SAS Institute Inc, CA). For behavioral data collected on lambs at age three weeks, the effects of day of the test, phase and their interactions were tested using the MIXED procedure (generalization of the standard linear model with REML (restricted maximum likelihood) and ML (maximum likelihood) estimation methods implemented with a Newton–Raphson algorithm; SAS Institute Inc., 1999) and with lamb as a random effect. The rearing pen and the position of the familiar caregiver in the test pen were initially included in the model but later removed due to the absence of any significant effect. We checked the condition of normality and homogeneity of the variance and the time spent near human was log transform. Post hoc comparisons after ANOVA were run using Tukey tests.

For HR parameters recorded on lambs at 8 weeks of age, we used the MIXED procedure for repeated data with an autoregressive (AR1) covariance structure of the R matrix to analyze the effect of the human presence or stroking: the effect of human (stroking *vs*. immobile), the effect of periods (4 periods of 2 min) and their interactions were tested on the cardiac activity of the lambs (HR, RMSSD and SDNN). The recording obtained on each variable (HR, RMSSD and SDNN) for each lamb when alone was used as co-variable in the model, and with lamb as a random effect. Post hoc comparisons after ANOVA were run using Least Square Differences (LSD). We checked the condition of normality and homogeneity of the variance.

For behavioral data of the 8 week old lambs, a Principal Component Analysis (Proc princomp procedure in SAS software) was first performed to take into account possible strong relationship between the different variables recorded during the 8 min, help the interpretation, and minimize the number of statistical tests needed. Due to the structure of their distribution, ear orientation was reorganized into three categories (% ears hanging scans, % ears axial scans, % ears in other postures). As no vocalization or escape attempt was recorded during this period, these variables were not factored in. Variables were interpreted according to their loadings on the most important components. The effect of human (stroking *vs*. immobile) on the two first components of the PCA was then compared using Student’s t-test.

Results are expressed as means ± SEM. Results with an associated probability less than or equal to 0.05 were considered significant.

## Results

### Affinity for the familiar caregiver by 3-week-old lambs: behavioral responses

Both for days 1 and 2, the lambs moved much less and vocalized much less when the familiar caregiver was present than when she was absent (Table 1). They also spent the majority of their time in the contact area where the familiar caregiver was crouching during the reunion phase (> 80%) than during the isolation phase (< 40%, Table 1). Day influenced lamb activity level and frequency of vocalizations, lambs were less active and less vocal on the second day, and we observed an interaction effect between day and phase of the test on vocalizations and number of zones crossed (Table 1).

**Table 1:** Behavioral outcomes of the human test (isolation-reunion-isolation phases) and effect of phases for the two consecutive test days (mean ± SEM) in 3-week-old lambs (n = 22).

	Day 1			Day 2			Effect of days		Effect of phases		Effect of interaction phases and days	
Phases	isolation	reunion	isolation	isolation	reunion	isolation	F_2,105_	P	F_1,105_	P	F_2,105_	P
Time spent in the contact area (s)	26.1 ± 3.8^a^	96.6 ± 7.3^b^	47.6 ± 3.7^c^	30.1 ± 4.6 ^a^	103.1 ± 7.7 ^b^	45 ± 6.7 ^c^	0.3	0.6	35.45	<0.001	1.41	0.25
Number of vocalizations	47.5 ± 2.3^a^	8.9 ± 1.9^b^	39.9 ± 2^c^	36.1 ± 1.8^d^	8.5 ± 1.5^b^	30.6 ± 1.7^f^	57.5	<0.001	123	<0.001	28.8	<0.001
Number of zones crossed	41.3 ± 2.8^a^	11.5 ± 2^b^	30.6 ± 3.3^c^	21.9 ± 2.5^d^	8.3 ± 1.2^e^	19.7 ± 3.3^d^	42	<0.001	69.4	<0.001	14.9	<0.001

In each line, values with different superscripts are significantly different (P < 0.05)

### Behavioral and cardiac responses to tactile contact by 8-week-old lambs

**Fig 3:**
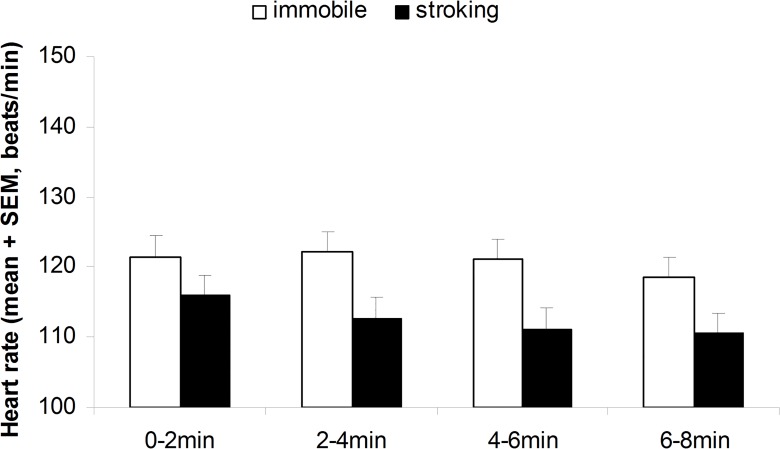
Heart rate recorded in the 8-week-old tested lambs during four successive 2-min periods of testing with a human stroking them (n = 11) or a human remaining passively immobile (n = 11). Human and periods effects P < 0.05.

**Fig 4:**
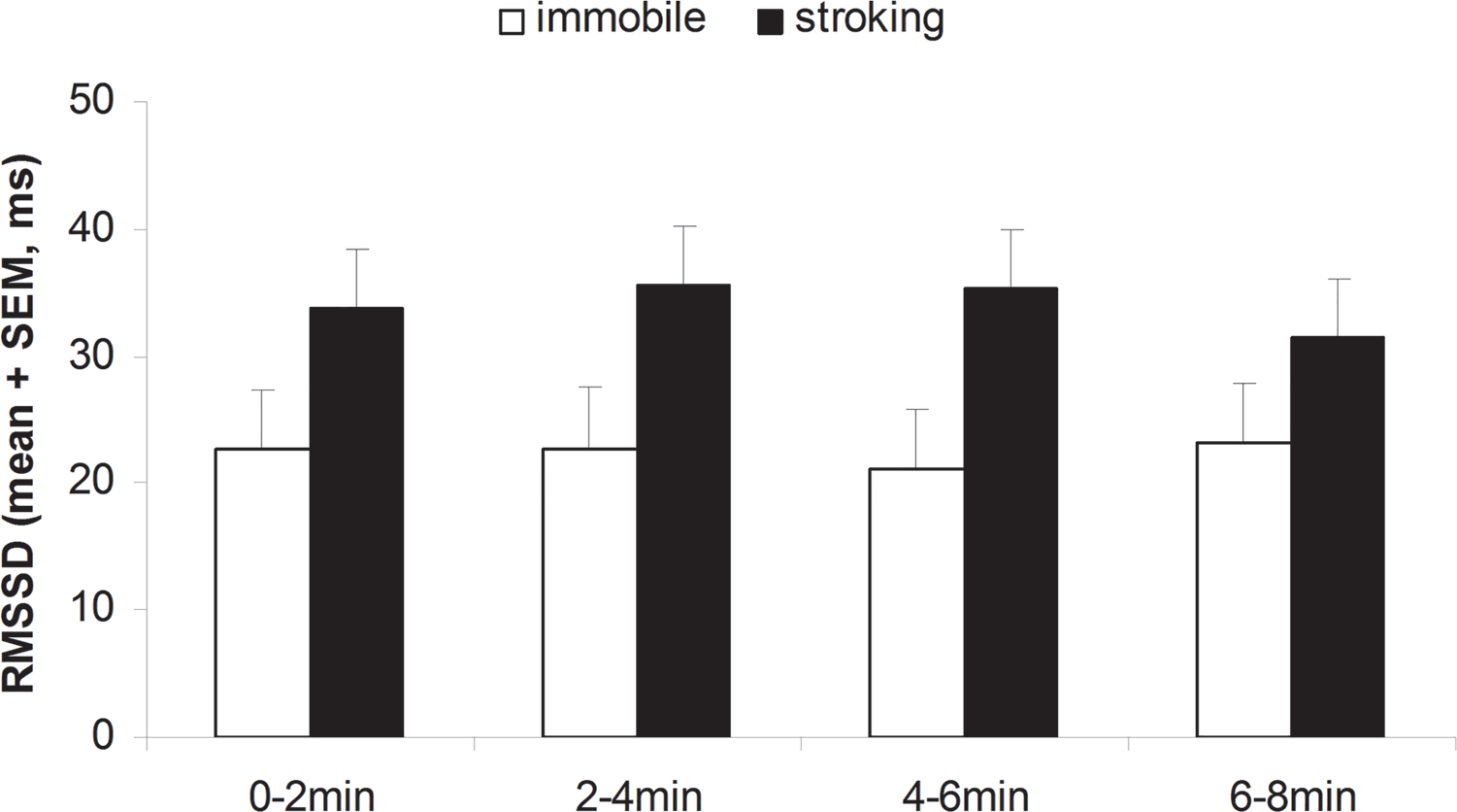
RMSSD recorded in the 8-week-old tested lambs during four successive 2-min periods of testing with a human stroking them (n = 11) or a human remaining passively immobile (n = 11). Human effect P < 0.05.

**Fig 5:**
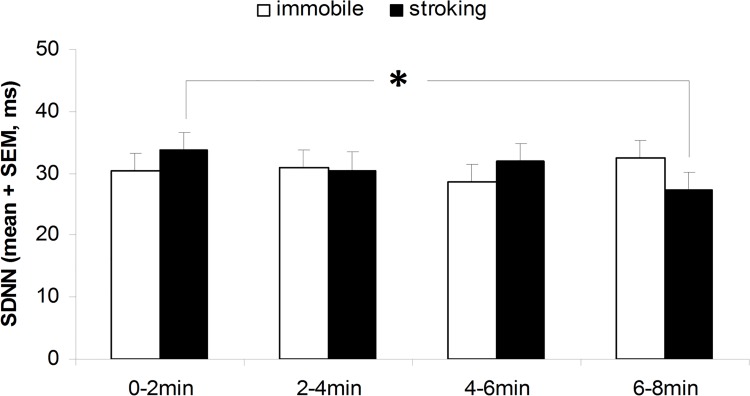
SDNN recorded in the 8-week-old tested lambs during four successive 2-min periods of testing with a human stroking them (n = 11) or a human remaining passively immobile (n = 11). * Human*periods effect P < 0.05.

**Cardiac activity.** Human tactile stimulation affected all cardiac parameters. Stroked lambs had a slower HR (F_1,60_ = 4.17; P = 0.046; Fig. 3) and higher RMSSD compared to unstroked lambs, without any significant change over the 8-min test duration (P > 0.25). Nevertheless HR of the stroked and unstroked lambs decreased with time (F_3,60_ = 2.95; P < 0.05; Fig. 4). There was no stroking or period effect (P > 0.7) on SDNN, but there was a significant interaction between stroking and period (F_3,60_ = 2.75; P = 0.05; Fig. 5), with SDNN decreasing significantly more between the first and last period in lambs being stroked (P < 0.05) compared to lambs left unstroked by the passive motionless human (P > 0.4).

**Fig 6:**
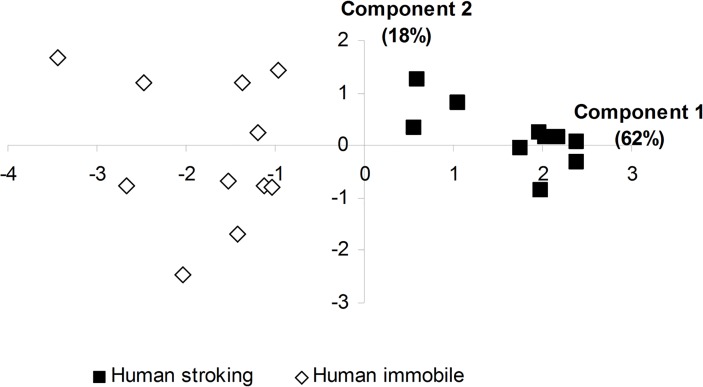
Distribution of the 8-week-old tested lambs on the two components of the PCA. Lambs were exposed to human stroking (n = 11) or just motionless human presence (n = 11) in the 8-min phase.

**Behavioral responses.** Lambs approached their familiar caregiver in 14.3 ± 4.7 sec when she entered the small pen. At this time, two lambs tried to escape and none of the lambs vocalized. Principal Component Analysis on behavioral variables recorded during the 8 min revealed that the first two components explained 80% of total variance, where the first component explained 62%. Load of each variable, indicating the variance explained by each component and with their eigenvalues are presented in Table 2. All variables are highly correlated with the first component (r_p_ > 0.6, P < 0.01). The more the animals stayed in contact with the human, the less they moved and the more their ears were hanging or in axial positions. The first component was interpreted as an evaluation of calmness of the animals while in contact with the human (Table 2). The lambs appeared much calmer during stroking than when left unstroked by the passive motionless human (t_20_ = 10.98; P < 0.001; Fig. 6).

**Table 2:** PCA using the six behavioral variables (observed in the 8-min phase of human stroking (n = 11) or motionless presence (n = 11)) recorded in lambs at 8 weeks of age.

Behavioral observations	Component 1	Component 2
percentage of time in contact with human	**0.41**	0.15
percentage of time on the knees of human	**0.45**	-0.06
percentage of ‘ears hanging’ observations	**0.47**	0.17
percentage of time moving	-0.4	**0.49**
percentage of ‘ears in axial posture’ observations	-0.38	**0.41**
percentage of ‘ears in other postures’ observations	-0.32	-**0.74**
Eigenvalue	3.76	1.07

Interpretable factor loadings are in bold. The first two components explained 80% of total variance, where the first component explained 62%.

## Discussion

In this study, gentle human tactile stimulation has been shown, for the first time to the author’s knowledge, to have a calming effect compared to simple passive human presence in species like sheep that do not show intraspecific grooming [12]. Lamb heart rate decreased continuously while in the presence of their caregiver. Lambs being stroked showed slower heart rate and higher parasympathetic activity (RMSSD) compared to lambs left unstroked. In addition, all behavioral variables were highly correlated with the main component axis of the PCA analyses: the more the animals stayed in contact with their caregiver, the less they moved and the more their ears were hanging. We will discuss how our results support the hypothesis that gentle physical contact with the caregiver is perceived positively by lambs, but also how we can soundly reject other potential explanatory mechanisms such as aversion to human contact, learned helplessness, habituation, or secondary association through other reinforcements such as food reward.

In the behavioral test at 3 weeks of age, when lambs were socially isolated, the presence of their familiar caregiver had a strong calming effect (less vocalization and locomotion with contact-seeking with the human). Our results are highly consistent with previous data from Tallet et al. [18,19] showing that artificially-reared lambs receiving daily human tactile contact expressed a stronger affinity for their caregiver than lambs receiving passive human presence or no exposure to human at all. Indeed in their experiment, handled and stroked lambs showed significantly higher motivation to seek the contact for their familiar caregiver than controls (human presence only). They also reacted more to his departure (more vocalizations) [18]. At 6 weeks of age, Coulon et al [24], studying lambs regularly stroked in a similar way, showed that behavioral responses by lambs to their familiar caregiver appear quite robust as, despite long periods of social isolation during the test, the lambs still spent more than 50% of the reunion time with the human. Long term (after weaning) affiliative responses towards the humans have been observed in lambs that received such type of contact in early age [17,32]

During the test on lambs’ behavior in the home pen, we observed what appears to be a calming effect of close proximity to a human: lamb heart rate decreased when a human was present. This decrease in HR has also been observed in other studies on human grooming of farm animals (e.g. in horses [13] and cattle [7]) and used as an indicator of positive emotion. Social separation and shearing the day before are stressful events for lambs that may have reinforced the calming effect observed in the close proximity to a human. It would so be interesting in a next experiment to study the effect of stroking according to the level of stress of the animals. In addition, stroked lambs in our experiment had a lower HR and a higher RMSSD than lambs exposed to motionless human presence. Previous studies showed that positive emotional states are characterized by slower HR and increased RMSSD compared to negative emotional states [22]. RMSSD is a good indicator of the parasympathetic (or vagal) branch that is predominant in resting condition. In addition, we also found that SDNN decreased significantly more between the first and last period in stroked lambs compared to lambs just in the presence of a motionless human, which further confirms the RMSSD results. Many previous studies have described an increase in sympathetic activity and a decrease in parasympathetic activity with emotional stress whereas positive emotions create increased parasympathetic activation (e.g. in humans: [33], in farm animals: [22]). Hence, we can confidently consider that lambs experienced positive emotions during stroking.

In addition, the behavioral data confirm the physiological outcome: the more animals stayed in contact with the human, the less they moved and the more their ears were hanging or in axial positions. This state is more clearly expressed during stroking than during passive human presence. A high proportion of axial or hanging ear postures has previously been observed in situations likely to induce positive emotional states, i.e. in cattle when fed fresh hay [7], and in sheep during stroking [22,23]. According to Boissy et al. [31], negative emotional experiences (suddenness, unfamiliarity, negative contrast, and uncontrollability) are associated with erected ears whereas positive emotional experiences could coincide with non-erected ears. In addition, the stroked lambs generally stayed on the caregiver’s knees without restraint (they were free to move away from the knees), which further suggests that stroking was effectively a positive experience: as proposed by Fraser and Duncan [34], the decision to keep accepting an action may be influenced by positive motivational affective state. Furthermore in our test situation, the lambs quickly approached the caregiver when he entered the pen and only two lambs tried to escape. Then over the 8 min of behavioral observation and whether the lambs had been stroked or not they showed no escape behavior and stayed on average 5 min in contact with their caregiver. This indicates that lambs were not afraid of their caregiver and that the human would be perceived positively. Indeed Destrez et al. [35] showed fear responses towards human in aversively treated lambs (high latency to approach, few contacts with human and high escape distance) compared to gently treated lambs. According to Tallet [18], the mere act of catching and holding seems rewarding for the lambs as the caregiver may, like the congeners in the home pen, provide the stimulations that the mother normally provides (helping regulate the body temperature).

To support our conclusion on a positive calming effect of stroking on artificially-reared lambs, we now discuss—and reasonably reject—alternative hypotheses to explain why the lambs appeared “calmer” even if the stroking procedure could be perceived as negative. The first alternative hypothesis to be refuted is the possibility that the observed “calming” effect reflects freezing or tonic immobility. The reduction of activity when lambs were caught and stroked could potentially be interpreted as an antipredator strategy seen in many animals when in danger, including artiodactyles [36,37]. Caro et al [36] reviewing such behavior in different species related it to hiding, with freezing enhancing crypticity. However, this is incompatible with the strong level of affinity expressed by lambs to their caregiver: lambs readily approached their familiar caregiver. Both in their home pen and in an arena test pen, they stayed in contact with her as it was previously shown in lambs where gentle interactions increased the animals’ motivation to interact with human [19,35]. The ear posture is also not tonic and alert but hanging [31].

A second hypothesis that needs to be rejected, with similar outcomes to above, is the fact that animals are simply habituated to human contact regardless of whether it is positive or negative. Habituation is defined as a “behavioral response decrement that results from repeated stimulation and that does not involve sensory adaptation, sensory fatigue or motor fatigue” [38]. An animal exposed to negative stimuli (e.g. electric shock, or negative social encounter) could also learn the futility of attempting to avoid an aversive event with potential later effects such as deficits in behavioral coping, associative learning, and emotional expression (collapsed under the term “learned helplessness” [39]). If the lambs were simply habituated to repeated human presence and contact, they should stop responding or respond only slightly to tactile stimulation. Consequently, in our testing procedure, they should have remained indifferent to their caregiver and not shown any change in presence or absence of the human or while being stroked. However, here, as in previous studies (e.g. [18,19,25]), artificially-reared lambs stroked daily for a few minutes expressed strong attraction for their familiar caretaker, not just during training sessions or in a unfamiliar arena test but also in the home pen test as evidenced by an immediate approach of the lambs. The position of their ears (hanging) and the parasympathetic cardiac activation reflecting positive emotion [31,23] argue against a simple habituation process or learned helplessness. To explore further the importance of experience of the human contact in the later response to humans, it will be useful in future work, to test the perception of gentle handling in regards to whether or not they have experienced it.

A last hypothesis—that we again dismiss here—is the possibility that artificially-fed lambs had been trained to feed artificially during their first two days and might then have associated all subsequent human contact with this early food training. If this was the case, later responses to humans would only be the result of a secondary reinforcement phenomenon through classical learning, and not the effect of stroking itself provided outside feeding sessions. However, we do not think this is the case. First, Tallet et al. [18] showed that holding lambs is sufficient to achieve the development of an affinity for a human caregiver. Secondly, training the lambs to suckle from the milk-feeder lasted for only the first two days of age and was performed without any stroking. In the three weeks of stroking sessions, stroking occurred at least half an hour before or after food was distributed precisely to avoid any association. Under these conditions, we would have expected an extinction of the early association between the human and the feeding reward.

The present work is a first step towards a deeper understanding of the perception of human stroking by lambs, and warrants further investigation. It is possible, for example, that providing regular simple physical contact is more relevant than the stroking *per se*. Indeed, Tallet et al [18] found that regularly catching artificially-reared lambs positively increased their approach behaviors to their familiar stockperson. Sheep are gregarious animals and spend a large amount of time in close physical proximity and physical contact [40]. In line with this hypothesis, in other species, skin-to-skin contact may create heart signal transference from one individual to another (e.g. in humans [41]), and the lamb HRs observed here could have harmonized with the HR of the familiar caregiver. Futures studies could investigate the effect of close human contact without stroking on heart rate variability. The effect of stroking could also vary according to physical (temperature, heart rate), emotional and social parameters (pressure, velocity). For example, human participants who received moderate-pressure massage exhibited a parasympathetic nervous system response while those who received light-pressure massage exhibited a sympathetic nervous system response [42] or when two friends held hands, their heart rates were influenced by the temperature of their hands, and the other feelings and bond between them [41]. Future studies could usefully investigate whether different stroking pressures from the caregiver’s hand have the same effect on lamb heart rate and whether the lambs react in the same way when stroked by a familiar or an unknown human or by an automated brush.

In conclusion, lambs that are accustomed to being stroked by their familiar caregiver show affiliative behavior to that person, relaxed ear postures, and the parasympathetic influence on their heart rate during stroking supports the hypothesis that they perceive it as positive. This study highlights that in species like sheep that do not show intraspecific grooming, providing tactile stimulation could be beneficial to the animal’s wellbeing.
